# Influence of Polymer Concentration and Nozzle Material on Centrifugal Fiber Spinning

**DOI:** 10.3390/polym12030575

**Published:** 2020-03-05

**Authors:** Jorgo Merchiers, Willem Meurs, Wim Deferme, Roos Peeters, Mieke Buntinx, Naveen K. Reddy

**Affiliations:** 1 Hasselt University, Institute for Materials Research (IMO-IMOMEC), B-3590 Diepenbeek, Belgium; jorgo.merchiers@uhasselt.be (J.M.); Willemmeurs@gmail.com (W.M.); wim.deferme@uhasselt.be (W.D.); roos.peeters@uhasselt.be (R.P.); mieke.buntinx@uhasselt.be (M.B.); 2 IMEC vzw-Division IMOMEC, Wetenschapspark 1, B-3590 Diepenbeek, Belgium

**Keywords:** centrifugal fiber spinning, nonwoven fibers, polymer rheology, wall slip

## Abstract

Centrifugal fiber spinning has recently emerged as a highly promising alternative technique for the production of nonwoven, ultrafine fiber mats. Due to its high production rate, it could provide a more technologically relevant fiber spinning technique than electrospinning. In this contribution, we examine the influence of polymer concentration and nozzle material on the centrifugal spinning process and the fiber morphology. We find that increasing the polymer concentration transforms the process from a beaded-fiber regime to a continuous-fiber regime. Furthermore, we find that not only fiber diameter is strongly concentration-dependent, but also the nozzle material plays a significant role, especially in the continuous-fiber regime. This was evaluated by the use of a polytetrafluoroethylene (PTFE) and an aluminum nozzle. We discuss the influence of polymer concentration on fiber morphology and show that the choice of nozzle material has a significant influence on the fiber diameter.

## 1. Introduction

Microfibers and nanofibers exhibit an extremely high surface-to-volume ratio, a low density, and a high pore volume [1,2,3]. These exceptional properties offer high added value to the material, making them suitable for a wide range of applications in the fields of environment [4,5,6], biomedical technology [7,8,9], and energy production [10,11,12,13,14]. Nonwoven fiber mats are generally obtained by the use of electrospinning, in which a polymeric fluid jet is pushed from a nozzle, where it is exposed to a high electric field. Here, the charged jet will be subjected to whipping and bending instabilities, leading to thinning of the jet and evaporation of the solvent [15,16,17]. However, due to a relatively low production rate (0.01–0.1 mL/min) [18,19] and the need for high voltages, the search for better alternative spinning techniques is necessary.

**Figure 1:**
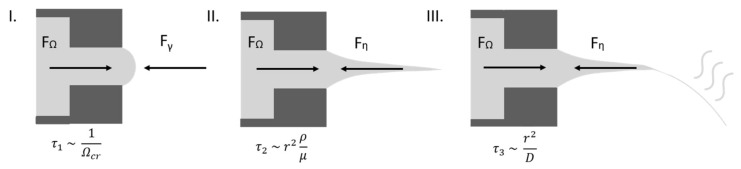
The three stages in centrifugal fiber spinning experiments, being the jet initiation (**I**), jet extension (**II**) and solvent evaporation (**III**), with their respective timescales.

Centrifugal fiber spinning can be considered as a promising alternative to conventional spinning techniques, such as electrospinning and melt-blow spinning. It is a very simple technique, based on centrifugal forces, and offers a higher production rate at a lower cost. This leads to a technology that is environmentally as well as economically advantageous over others. This highly versatile method was first developed by the FibeRio Technology Corporation and introduced as the Forcespinning Technology^®^ [20]. Subsequent experimental and theoretical studies revealed that the process involves three stages, schematically depicted in Figure 1. These are: the jet initiation stage (I), the jet-elongation stage (II), and the solvent evaporation stage (III), each characterized by a proper timescale. The characteristic time for the jet-initiation stage, τ_1_, is proportional to the inverse of the critical rotational speed, Ω_cr_. Here the centrifugal force must overcome the surface tension at the nozzle tip to initiate jet formation. In the jet-elongation stage, the viscous forces counteract the centrifugal forces. Here the characteristic timescale, τ_2_, is mainly determined by the shear and extensional viscosity, as well as the density (ρ) of the polymer solution. Finally, the third characteristic timescale, τ_3_, is determined by the diffusion coefficient (D), of the solvent through the drying polymer with jet radius, r [21,22,23,24,25]. According to several published studies, the fiber morphology depends on the polymer concentration, molecular weight, centrifugal speed, collector distance, and nozzle diameter [26,27,28,29]. Even though the centrifugal spinning technique has attracted a lot of interest, and the number of publications has increased tremendously due to its simplicity and its ability to produce fibers at a large scale, control over the fiber morphology remains the major limitation to upscaling [29,30,31,32,33,34,35,36,37,38,39,40,41,42,43,44,45]. 

In this contribution, we investigate the role of both concentration-dependent variation in shear viscosity and the choice of nozzle material on the fiber morphology. Although most of the literature on the centrifugal spinning technique focuses on variations in rotational speed, nozzle diameter, and collector distance, we seek to determine whether certain events inside the centrifuge nozzle influence the fiber morphology, i.e., average fiber diameter, diameter distribution, and bead formation. We provide results from a systematic study of the effect of polymer concentration and nozzle material on the fiber morphology and average diameter by means of a home-built centrifugal spinning setup. We experimentally show the influence of polymer concentration on the fiber morphology. The focus was set on the concentration range where there is a transition from bead-on-string to continuous fiber morphology. We show that a smooth morphology transition occurs as a function of polymer concentration from beads-on-string to continuous fibers, but when plotted as a function of fiber diameter the same transition is very sharp, showing two distinct slopes. Finally, we show that the choice of nozzle material can be considered as a novel parameter that has a significant influence on the outcome of the centrifugal spinning process. By comparing the morphological results of fibers spun from two different nozzle materials, a significant difference in concentration dependence was observed. 

## 2. Materials and Methods

### 2.1. Materials

Polystyrene (PS, M_W_ = 190 kg/mol) and tetrahydrofuran (THF, AnalaR NORMAPUR) were purchased from VWR Chemicals (Oud-Heverlee, Belgium) and used without further purification. Polymer solutions were prepared by slowly adding the polymer to the solvent under continuous magnetic stirring. Homogeneous mixing was ensured by stirring for around 24 h in hermetically sealed vials. A concentration range of 10.0–25.0 wt %, in steps of 2.5 wt %, was prepared.

### 2.2. Rheological Characterization

The shear viscosity of the different PS solutions was measured using an ARG2 stress-controlled rheometer (TA Instruments, New Castle, DE, USA), with cone-and-plate geometry (40 mm diameter, 1° cone angle). The temperature was fixed at 25 °C using a Peltier element and a solvent trap was used to reduce evaporation. Steady-state viscosity was recorded for shear stress values ranging from 0.01 to 10 Pa within the experimentally accessible window of the rheometer. A steady-state tolerance of 3% was applied to each measurement. 

### 2.3. Fiber Characterization

The morphology of the PS fibers was analyzed using a Hitachi TM3000 tabletop scanning electron microscope (SEM) (Hitachi, Tokyo, Japan). Samples were imaged at 15 kV with a 5 mm working distance. The image analysis was carried out by means of ImageJ software (Bethesda, MD, USA). Sampling of the fibers was done in such a way that at least 100 fibers were measured, evenly divided over at least three different SEM images, which were taken at random spots on each sample. This was done to obtain statistically reliable fiber diameters. The fiber diameter distributions are presented together with the SEM images in this paper.

### 2.4. Contact Angle

THF was used as a testing liquid, and was deposited as 2-µL droplets by a Hamilton syringe onto the substrates (Aluminum and PTFE). A software-controlled needle dosing system (DSA 100E, KRÜSS GmbH, Hamburg, Germany) was used for static sessile drop contact angle measurements. The contact angles were measured after stabilization of the drop shape (typically after 5 s) and are reported as an average of five measurements.

### 2.5. Force to Push Polymer Solution from Different Nozzle Materials

The force measurements were performed using an MTS Systems (Adamel Lhomargy tensile tester, Roissy-en-Brie, France) at a crosshead speed of 500 mm/min. For these experiments, a 10-mL glass syringe was used, on which both the aluminum and PTFE nozzles, used to obtain PS fibers, could be fitted via a metal Luer lock connection. The polymer solutions were pushed at a controlled flow rate of 75 mL/min through the nozzle. A set of three measurements was performed for each solution.

## 3. Results

### 3.1. Fiber Spinning Setup

**Figure 2:**
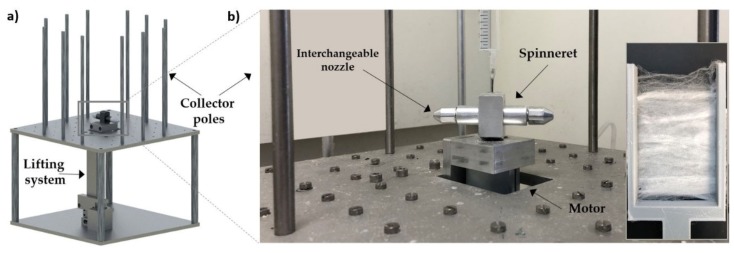
(**a**) Schematic view of the fiber spinning centrifuge with lift system. (**b**) The fiber spinneret with an interchangeable nozzle in which the polymer solution is added via the syringe on top (which is connected to a syringe pump to control the solution flow rate). The inset in (**b**) shows a macroscopic picture of the aligned fibers.

A home-built centrifugal spinning setup with an arm-style spinneret and a novel lifting system was developed as shown in Figure 2. With this system, the stacking of fibers on top of each other could be reduced, leading to more open and aligned fiber mats. Polymer solution is added via a syringe pump to the rotating spinneret at a constant flow rate of 1 mL/min. The flow rate at which the polymer jet is ejected is controlled by the stress at the nozzle tip due to centrifugal force. The flow rate of 1 mL/min was empirically determined to be sufficient to have a continuous flow of polymer solution within the nozzle. The spinneret was equipped with a screw thread to interchange different types of nozzle tips as needed. Nozzle tips with a nozzle diameter of 0.6 mm were fabricated in both aluminum and PTFE.

The collector poles were set 12 cm from the nozzle tip and fibers were produced at a rotation speed of 4000 rpm. Furthermore, fiber spinning experiments were always carried out at room temperature (22 °C) and at a relative humidity of approximately 40%. (A detailed sketch of the spinneret design is provided in Figure S1.)

### 3.2. Characterization of Polymer Solutions

**Figure 3:**
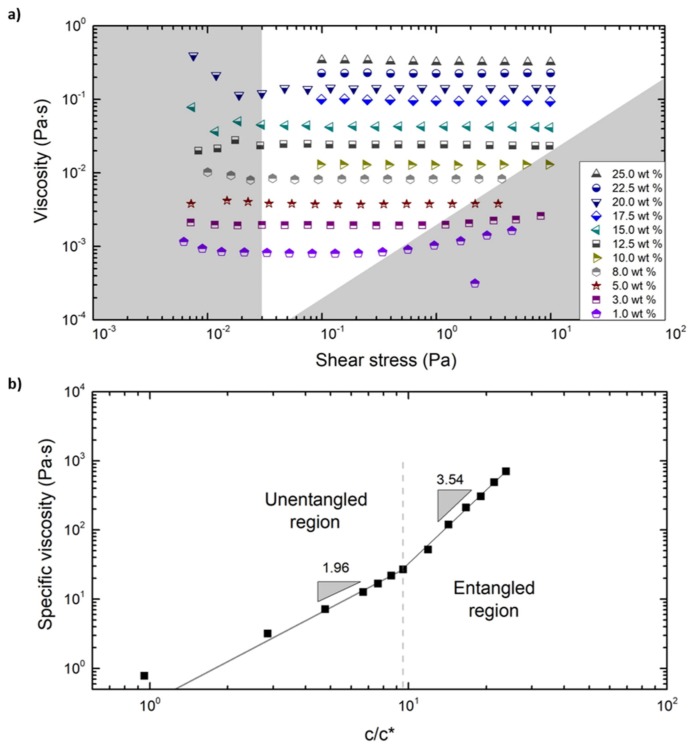
(**a**) Steady shear viscosity as a function of shear stress. The region between the gray zones is the accessible window of the rheometer. (**b**) Specific viscosity as a function of PS concentration normalized with overlap concentration (c*).

The steady shear viscosity measurements were carried out for a wide range of concentrations ranging from 1.0 to 25.0 wt %, as shown in Figure 3a. The viscosity measurements were carried out for a range of shear stresses set by the accessible torque range of the rheometer. The limits of the instrument were considered to avoid erroneous data, and are depicted by the gray zones in Figure 3a. 

The low torque limit was determined via [46]

(1) $$\eta >\frac{F_{\tau }T_{\min}}{\dot{\gamma }},$$

where η is the shear viscosity and $\dot{\gamma }$ is the shear rate.

Furthermore, F_τ_ = 3/(2πR^3^) with measuring geometry radius, R = 20 mm, and minimum measurable torque, T_min_ = 0.1 μN·m. At higher shear rates, the limit is set by secondary flow in the sample due to inertia [47,48], and is given by

(2) η>L3/RRecritργ˙,

where the critical Reynolds number Re_crit_ = 4, the maximum gap between the cone edge and plate is L = 0.7 mm and the density of THF ρ = 889 kg/m^3^. 

The specific viscosity at each concentration was calculated by the following equation [48]:

(3) $$\eta_{\mathrm{sp}}=\frac{\eta_{0}-\eta_{s}}{\eta_{s}},$$

where η_0_ is the zero-shear viscosity determined for every concentration from Figure 3a and η_s_ is the solvent viscosity = 0.46 mPa·s. The critical overlap concentration (c*) of the PS chains in THF was estimated by the following equation [49]:

(4) $$c^{*}=\frac{0.77}{\left[\eta \right]},$$

in which [η] is the intrinsic viscosity. The critical overlap concentration, c*, was determined to be 0.012 g/mL using Equation (4), through which the intrinsic viscosity, [η] = 65.24 mL/g, was experimentally determined via the Mark‒Houwink equation using gel permeation chromatography (GPC). The Mark‒Houwink equation is:

(5) $$\left[\eta \right]=K\times M_{V}^{\alpha },$$

in which K and α are the Mark‒Houwink parameters, determined to be 0.0141 mL/g and 0.70, respectively. The experimentally determined viscosity averaged molecular weight (by GPC), 171.9 kg/mol, was used to calculate the intrinsic viscosity. The intrinsic viscosity obtained here is similar to the values found in the literature for PS in a good solvent [50,51]. Previous studies on electrospinning show that topological interactions become relevant above the critical entanglement concentration, c_e_ ≈ 10c* for flexible polymers [52,53,54,55,56]. In the case of narrow disperse samples (without high M_w_ content), the solution with a concentration below c* does not possess the required viscoelastic forces to overcome the instabilities that are exerted on the liquid jet, leading to jet breakup and bead formation. In the case of disperse samples, the influence of the high M_w_ content in the sample will change the viscoelastic response of the polymers. This will cause a significant decrease in the critical concentration at which fibers can be spun, leading to jet breakup and drop formation at lower concentrations than the predicted critical values [26,27,49,52,54].

Figure 3b shows a plot of the specific viscosity, η_sp_, as a function of the polymer concentration normalized by the overlap concentration, c/c*. Since the 1 wt % (= 0.011 g/mL) solution lies below the c* (= 0.012 g/mL), this point will not correspond with the power law exponent and therefore deviates significantly from the fitted line. The entanglement concentration, c_e_, was determined as the point where an abrupt change in the power law relation between both variables occurred. The power law exponent increased from 1.96 to 3.54 at a rescaled concentration value of c/c* = 9.53, which corresponds to the entanglement concentration of c_e_ ≈ 0.112 g/mL (= 10.0 wt %). 

This is in agreement with literature values, where the power law relations of an unentangled polymer solution are η_sp_ ∝ (c/c*)^2^ and for an entangled polymer solution η_sp_ ∝ (c/c*)^3.9^ in a good solvent [57,58]. In other words, beyond c_e_ the change in viscosity becomes more sensitive to changes in polymer concentration. All the centrifugal fiber spinning experiments were therefore conducted with polymer solutions starting at this critical concentration of 10.0 wt %. Zero shear viscosity and specific viscosity at various polymer concentrations are also presented in Table 1. 

### 3.3. Changes to Fiber Morphology with Polymer Concentration

**Figure 4:**
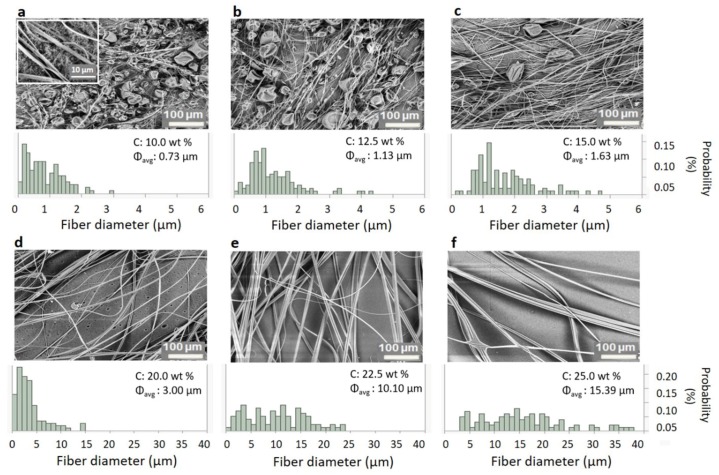
SEM images of fibers spun from the aluminum nozzle at different polymer concentrations and their corresponding diameter distribution. Inset in (**a**) is an enlargement (30 µm image width) showing the presence of fibers. (Note: the fiber diameter on the x-axis is from 0 to 6 µm for (**a**–**c**), and from 0 to 40 µm for (**d**–**f**)).

Figure 4 shows the SEM images of the fiber morphology obtained for a range of different concentrations of PS in THF, together with the fiber diameter distributions. To prevent the formation of fibers at lower rotational speeds during startup, the spinneret was started and left spinning until it reached 4000 rpm before dispensing the polymer solution. Below a polymer concentration of 10.0 wt %, only beads were formed due to the lack of sufficient viscoelastic stresses required to prevent capillary breakup [52]. The experimental lower limit of 10.0 wt % to form fibers agrees with the entanglement concentration (c_e_ ≈ 10 c*), as calculated before from the overlap concentration (c*). Above 25.0 wt %, the solution’s viscosity was too high for the centrifugal force to overcome the viscous force. 

The fiber diameter and its distribution (in terms of standard deviation) for the intermediate concentrations obtained using the aluminum nozzle are shown in Table 1. The tabulated values were determined by averaging at least 100 fibers in the sample. In Figure 4, two phenomena can be observed upon increasing the polymer concentration: 1) Beads-on-a-String (BOAS) morphology with a slow increase in fiber diameter at lower concentrations, which is shown in Figure 4a–c (from 10.0 to 15.0 wt %), and 2) bead-free fiber morphology with a sharp increase in diameter for higher concentrations, which is shown in Figure 4d–f (>17.5 wt %). 

**Figure 5:**
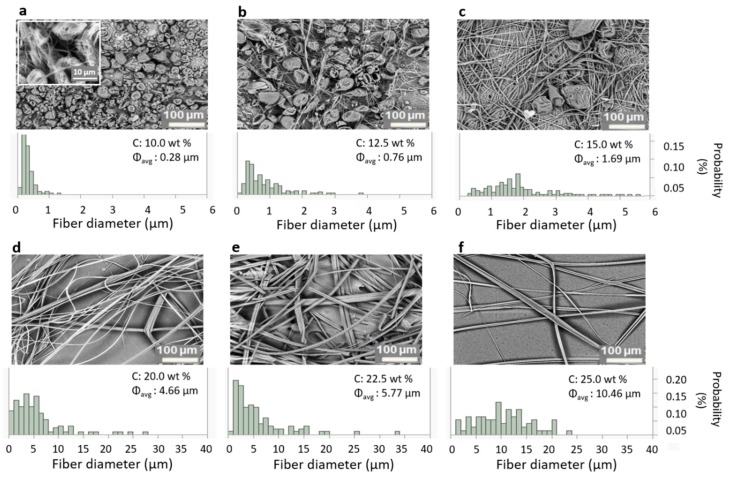
SEM images of fibers spun from the PTFE nozzle at different polymer concentrations and their corresponding diameter distribution. The inset in (**a**) is an enlargement (30 µm image width) showing the presence of fibers. (Note: the fiber diameter scale is from 0 to 6 µm for (**a**–**c**), and from 0 to 40 µm for (**d**–**f**)).

Fiber spinning experiments of the same polymer solutions, spun from a PTFE nozzle with exactly the same dimensions, are shown in Figure 5. The results from these experiments are also listed in Table 1, from which a similar trend as with the aluminum nozzle was observed for the two concentration regions: a slow increase in fiber diameter in the presence of beads for the lower polymer concentrations, and a fast increase in fiber diameter with no beads at higher polymer concentrations. Also, the transition from beaded fibers to continuous fibers occurred in the same region as it did for the aluminum nozzle. The fiber morphology using PTFE is similar to what has been seen in the literature [59,60,61]. However, a significant difference was observed in the dependence of the fiber diameter on the polymer concentration between the fibers spun from both nozzles as shown in Table 1. 

## 4. Discussion

### 4.1. Fiber Morphology

**BOAS morphology:** For concentrations close to c_e_, the polymer jets’ viscoelastic stress is sufficient to withstand the capillary breakup, leading to fiber formation. At this transition concentration, the capillary instabilities will be significant when the polymer jet is still thick, but as the jet thins the viscoelastic stress becomes significant due to the high extensional rates. This event will eventually lead to the BOAS morphology [62,63,64]. As most of the bulk polymer is in the bead part, the string connecting the beads will drain into the beads, thinning the string further [65,66]. This process, in combination with solvent evaporation, leads to extremely thin solid fibers whenever there is a BOAS morphology. As the concentration is increased, the fiber diameter increases slowly, reducing the size and number of the beads (see Figure 4 and Figure 5a–c).

**Continuous fiber morphology:** In Figure 4 and Figure 5d–f, no more beads were observed for solutions of concentrations > 17.5 wt %. In these solutions, the viscoelastic forces are strong enough to counteract capillary breakup to avoid the formation of beads. This will lead to a faster increase of the fiber diameter upon further increase of concentration, as the bulk material must stay in the fiber—unlike for lower concentrations, where it could drain into the beads. Additionally, other factors like the increase in viscosity and solvent evaporation (which leads to skin formation on the polymer jets) also come into play [39]. 

### 4.2. Polymer Solution‒Wall Interactions

One of the events that plays a role inside the nozzle is the interaction between the polymer solution and the nozzle wall [67,68,69,70,71,72]. Unlike in electrospinning, where the polymer solution is dispensed at extremely low flow rates, in centrifugal spinning the flow rates are much higher, leading to high shear rates within the nozzle. In general, the no-slip boundary condition at the nozzle wall is assumed in the bulk flow of fluids. However, this no-slip boundary condition will not be valid for many complex fluids, such as polymer solutions. Especially at higher polymer concentrations things become even more complicated when a thin polymer-depleted layer of much lower viscosity than the bulk solution forms at the solid boundary, in which the surface wettability plays a crucial role [73,74,75,76,77]. One of the open questions in centrifugal spinning that has not been dealt with in detail, to our knowledge, is the effect of nozzle material on the final fiber morphology. 

**Figure 6:**
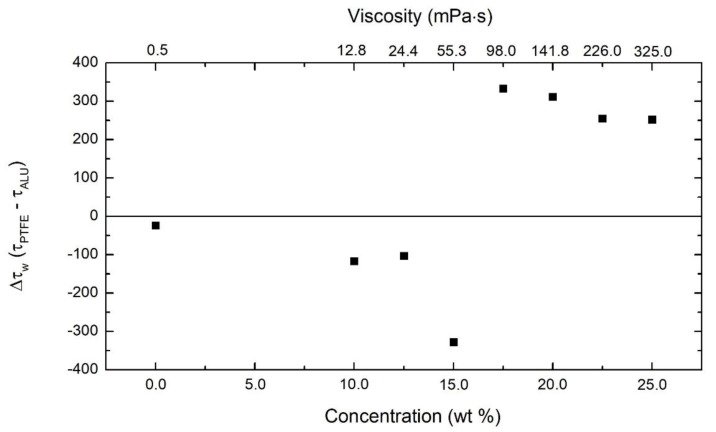
The difference in stress needed to push the polymer solution through PTFE and aluminum nozzles, respectively, as a function of the polymer concentration.

The contact angle, which is a measure of wettability, was determined using the solvent (THF) on both an aluminum (16°) and a PTFE (25°) substrate. These low contact angles indicate that the interfacial tension between solvent and nozzle is small, leading to the no-slip boundary condition for the pure solvent. However, to assess the presence of slip within the nozzle during flow, we directly measured the force required to push the polymer solution out of the two nozzles at a fixed flow rate. A lower force needed to push the solution implies relatively more wall slip as compared to a higher force. Figure 6 shows the difference in stress (force/nozzle cross section area) required for the flow of the polymer solutions through the PTFE and the aluminum nozzle. The difference between the stress needed to push pure THF through both nozzles is similar, with a slightly negative value, meaning that it takes marginally more force to push THF through the aluminum nozzle than it does through the PTFE nozzle, in line with the measured contact angles. 

The same experiments were conducted for the polymer solutions that were used in the fiber spinning experiments. The relative difference between stress values was also slightly negative for the low concentrated solutions (<15.0 wt %), indicating that no significant interactions exist between wall and solution (low-concentration polymer solutions behave similarly to pure THF). For intermediate concentrations (around 15.0 wt %), the stress difference shows a steep increase from the lower concentrations, which would imply that there is more slip occurring in the PTFE nozzle. Even though a higher stress difference was seen at this intermediate concentration, there was no sudden change seen in fiber diameter. Currently, no explanation can be provided for this behavior. Once the concentration enters the highly concentrated regime (≥17.5 wt %) where solely continuous fibers were formed, the stress difference suddenly becomes positive, meaning that the force required to push the polymer solution through the PTFE nozzle is higher than that for the aluminum nozzle. This sudden change correlates with the faster increase in fiber diameter for the high concentration regime for fibers spun from the aluminum nozzle as compared to the PTFE one, as tabulated in Table 1. We believe that the reason for this sudden change in force between the nozzles can be assigned to the onset of the ‘apparent’ wall slip, which occurs to a higher extent in the aluminum nozzle compared to that of PTFE for polymer solutions in THF. The ‘apparent’ wall slip causes an easier flow, which induces a lower force needed to push the solution in the case of the aluminum nozzle, changing the flow profile in the nozzle into a plug flow. As this plug flow polymer solution is pulled due to centrifugal force, the jet’s starting diameter will be large, leading to thicker fibers. In the case of the PTFE nozzle, the higher force required to push the polymer solution implies no or relatively low ‘apparent’ wall slip, making the flow profile closer to parabolic. This fluid with parabolic flow profile when pulled out of the nozzle leads to a smaller initial jet diameter, which leads to fibers with smaller diameter as compared to fibers produced from the aluminum nozzle. 

**Figure 7:**
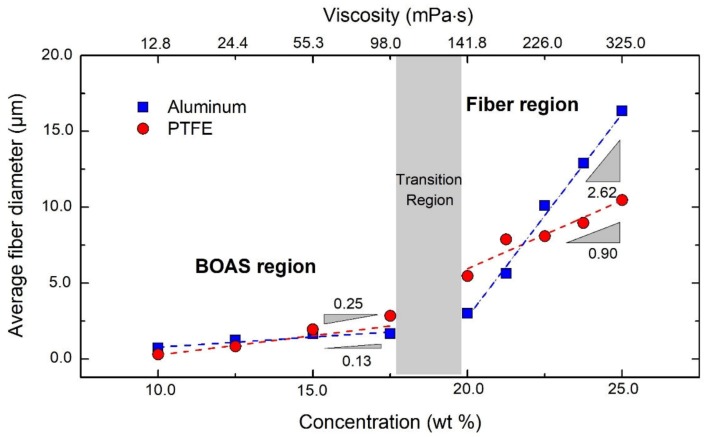
Change in fiber diameter as a function of polymer concentration for fibers spun using aluminum and PTFE nozzles. The error bars represent the standard deviations of the fiber diameters. Also given in the top x-axis is the shear viscosity for each polymer concentration.

In Figure 7, the average fiber diameters for the samples spun with both the aluminum (squares) and the PTFE nozzle (circles) is plotted as a function of the polymer concentration, showing that the influence of polymer concentration on the fiber diameter is different for both nozzles. In the lower polymer concentration region, the diameter of the fibers spun from the PTFE nozzle increases with concentration (with a slope = 0.25), almost similar to that spun from the aluminum nozzle (slope = 0.13). In the higher-concentration region, the diameter of the fibers spun from the aluminum nozzle increases significantly faster (slope = 2.62) than it does for the PTFE nozzle (slope = 0.90). 

This means that during the transition from a low to a high concentration region, the change in diameter is significantly less pronounced for fibers produced from the PTFE nozzle than from aluminum nozzle. The concentration region in which this increase in fiber diameter sets in is referred to as the transition region in Figure 7, within the gray zone, where the slopes of the two concentration regions intersect. This transition region also marks the change in fiber morphology from BOAS to bead-free fibers. The difference between the nozzle types shows that not only the polymer concentration (and therefore the viscosity) but also the nozzle material plays a significant role in the centrifugal fiber spinning process. In other words, the interaction of the polymer solution with the nozzle walls is of importance in order to be able to control the centrifuge fiber spinning process due to a significant flow inside the nozzles, as compared to other techniques where the flow rates are minimal. 

## 5. Conclusions

In this paper, we experimentally determined the influence of polymer concentration and nozzle material on the fiber diameter and morphology using a home-built centrifugal spinning setup. Viscosity measurements were performed on a wide range of PS solutions, ranging from 1.0 to 25.0 wt %. The rheological measurements led to a transition of the power law exponent from 1.96 to 3.54, for the relationship between the specific viscosity and the normalized concentration. This transition corresponds to the change from an unentangled polymer system to an entangled one, and occurred at c_e_ ≈ 10 wt %. From this concentration, the fiber spinning experiments were subsequently examined as a function of the polymer concentration up until 25.0 wt %. These experiments revealed a morphological transition in the fiber samples from the beads-on-string morphology, for the lower-concentration region (<17.5 wt %), to a continuous-fiber morphology, for the higher-concentration region (>17.5 wt %). This morphological transition was accompanied by a significant change in the slope of the fiber diameter dependence on polymer concentration and therefore on solution viscosity. 

Furthermore, this change was shown to be more significant for fibers produced from an aluminum nozzle, as compared to a PTFE nozzle having identical dimensions. This difference is attributed to the presence of a higher apparent wall slip in the case of an aluminum nozzle at the higher polymer concentrations, leading to a ‘plug’ flow through the nozzle and, subsequently, producing thicker fibers. The apparent wall slip behavior was shown to be present by measuring the difference between the force needed to push the solutions through the PTFE and the aluminum nozzles. This result led to the conclusion that, beyond a critical concentration, the polymer‒nozzle wall interaction becomes significant and must be considered during fiber production, using centrifugal spinning and other spinning processes where there is a significant flow of polymer solution inside the nozzle.

## Figures and Tables

**Table 1:** Experimentally determined values of the zero-shear viscosity (η_0_) and specific viscosity (η_sp_) of PS solutions, diameter (Φ) and standard deviation (SD) of polymer fibers obtained for each PS concentration using aluminum (ALU) and PTFE nozzles.

c (wt %)	η_0_ (Pa∙s)	η_sp_ (-)	Φ (ALU) (µm)	SD (µm)	Φ (PTFE) (µm)	SD (µm)
10.0	0.013	26.8	0.73	0.58	0.28	0.19
12.5	0.024	52.0	1.13	0.77	0.76	0.63
15.0	0.055	119.2	1.63	0.88	1.69	1.29
17.5	0.098	212.0	1.68	1.19	2.46	2.30
20.0	0.142	307.3	3.00	2.94	5.42	4.70
22.5	0.226	490.3	10.10	5.79	8.08	5.44
25.0	0.325	705.5	15.39	10.05	10.46	5.16

## Supplementary Materials

The following are available online at https://www.mdpi.com/2073-4360/12/3/575/s1, Figure S1: Exploded view of the assembly of the centrifugal fiber spinning setup.
