# Supplementary figures and images for: Influence of Polymer Concentration and Nozzle Material on Centrifugal Fiber Spinning
Source: Polymers (Basel). 2020 Mar 5;12(3):575. doi: 10.3390/polym12030575 (PMC7182933; doi:10.3390/polym12030575)

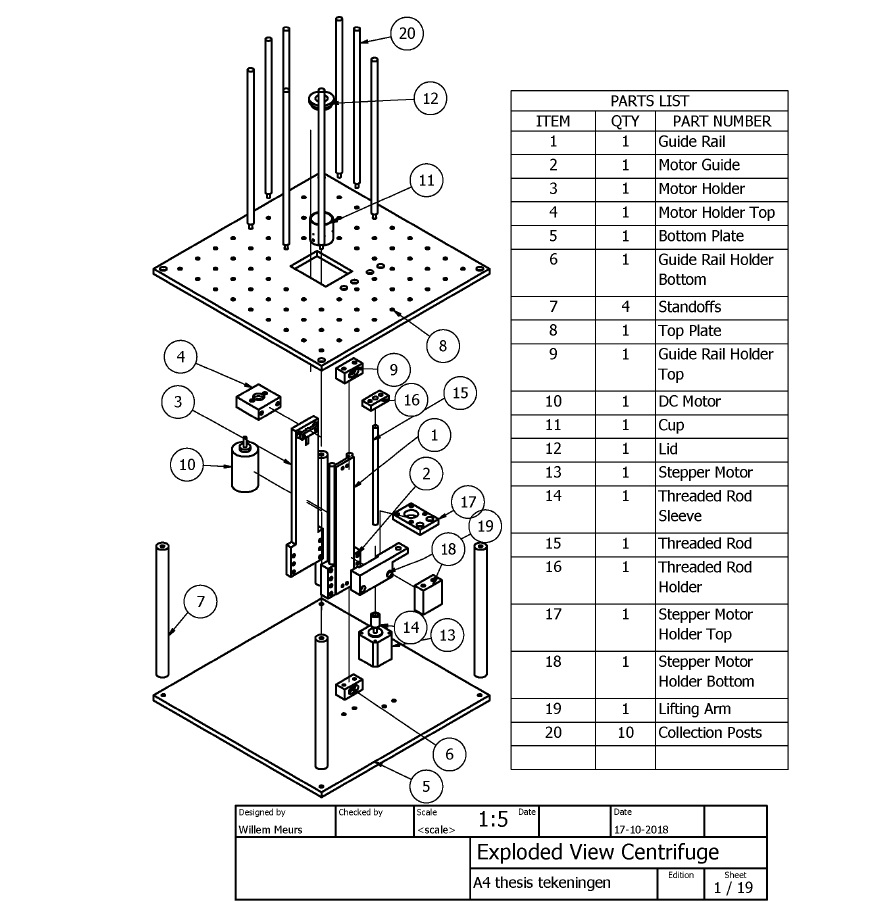

Supplement: Supplementary file 1 [file polymers-12-00575-s001.zip › polymers-727531-SI.jpg]
